# Metformin treatment ameliorates diabetes‐associated decline in hippocampal neurogenesis and memory via phosphorylation of insulin receptor substrate 1

**DOI:** 10.1002/2211-5463.12436

**Published:** 2018-05-18

**Authors:** Daisuke Tanokashira, Eiko Kurata, Wataru Fukuokaya, Kenshiro Kawabe, Mana Kashiwada, Hideyuki Takeuchi, Masamitsu Nakazato, Akiko Taguchi

**Affiliations:** ^1^ Department of Integrative Aging Neuroscience National Center for Geriatrics and Gerontology Obu Japan; ^2^ Department of Neurology, Respirology, Endocrinology and Metabolism Miyazaki University School of Medicine Japan; ^3^ Department of Neurology and Stroke Medicine Yokohama City University Graduate School of Medicine Japan

**Keywords:** dementia, diabetes, diabetic drugs, drug repositioning, IRS1 serine phosphorylation, memory function

## Abstract

Age‐related reduction in adult hippocampal neurogenesis is correlated with cognitive impairment. Diabetes is a chronic systemic disease that negatively affects adult neural stem cells and memory functions in the hippocampus. Despite growing concern regarding the potential role of diabetic drugs in neural abnormalities, their effects on progressive deterioration of neurogenesis and cognitive functions remain unknown. Here, we show that the combination of aging and diabetes in mice causes a marked decrease in hippocampal neurogenesis along with memory impairment and elevated neuroinflammation. Prolonged treatment with metformin, a biguanide antidiabetic medication, promotes cell proliferation and neuronal differentiation and inhibits aging‐ and diabetes‐associated microglial activation, which is related to homeostatic neurogenesis, leading to enhanced hippocampal neurogenesis in middle‐aged diabetic mice. Although chronic therapy with metformin fails to achieve recovery from hyperglycemia, a key feature of diabetes in middle‐aged diabetic mice, it improves hippocampal‐dependent spatial memory functions accompanied by increased phosphorylation of adenosine monophosphate‐activated protein kinase (AMPK), atypical protein kinase C ζ (aPKC ζ), and insulin receptor substrate 1 (IRS1) at selective serine residues in the hippocampus. Our findings suggest that signaling networks acting through long‐term metformin‐stimulated phosphorylation of AMPK, aPKC ζ/λ, and IRS1 serine sites contribute to neuroprotective effects on hippocampal neurogenesis and cognitive function independent of a hypoglycemic effect.

**Abbreviations**
AD: Alzheimer's disease
AMP: adenosine monophosphate
AMPK: AMP‐activated protein kinase
aPKC: atypical protein kinase C
DG: dentate gyrus
DIO: diet‐induced obesity
FFA: free fatty acid
GCL: granule cell layer
HD: Huntington's disease
HDL‐C: HDL cholesterol
HDL: high‐density lipoprotein
HFD: high‐fat‐diet
IGF: insulin‐like growth factor
IR: insulin receptor
IRS: insulin receptor substrate
LDL‐C: LDL cholesterol
LDL: low‐density lipoprotein
mTOR: mammalian target of rapamycin
T‐CHO: total cholesterol
TG: triglycerol

Adult neurogenesis occurs throughout life in specific brain regions of mammals, including the hippocampus and subventricular zone 1. Alterations in hippocampal neurogenesis are associated with neurological and psychiatric disorders, such as epilepsy, Alzheimer's disease (AD), Parkinson's disease, Huntington's disease (HD), and depression 2. In addition, the systemic milieu in the context of aging leads to a decline in hippocampal neurogenesis and cognitive functions in animals 3, which also occurs in model animals with type 1 or type 2 diabetes 4, 5, such as streptozotocin‐induced type 1 diabetes model and high‐fat‐diet (HFD)‐induced type 2 diabetes (diet‐induced obesity: DIO) model animals 4, 5, 6, 7, 8.

Accumulating evidence shows that diabetic therapies may improve cognitive functions in model animals or humans with diabetes 9, 10, 11, 12, 13, 14. For example, treatment with pioglitazone, a thiazolidine‐based diabetic drug, reduces the risk of dementia in patients with diabetes and improves both glucose metabolism and memory functions in patients with AD and diabetes 9. Similar finding has been obtained for therapy with metformin, a biguanide antidiabetic medication, and the first‐line drug for type 2 diabetes. Metformin lowers blood glucose levels by decreasing basal hepatic glucose output and increasing glucose uptake by skeletal muscle through the activation of the adenosine monophosphate (AMP)‐activated protein kinase (AMPK), an effector of metformin, and inhibition of the mammalian target of rapamycin (mTOR) pathway 15, 16, 17, 18, 19, 20. It also reduces cognitive decline and the risk of dementia in patients with type 2 diabetes compared with that in patients with type 2 diabetes without medication 10, 12, 15, suggesting that metformin exerts neuroprotective effects against cognitive dysfunction. The administration of metformin improves cognitive performance or hippocampal neurogenesis in chemical‐induced neurodegenerative model animals 21, 22. However, the effect of long‐term treatment with metformin on spatial memory functions and adult hippocampal neurogenesis in mice with combination of aging and diabetes (middle‐aged DIO mice) remains unknown.

Here, we show that chronic administration of metformin facilitates cell proliferation and neuronal differentiation and inhibits aging‐ and diabetes‐related neuroinflammation in the dentate gyrus (DG) of middle‐aged DIO mice, leading to increased hippocampal neurogenesis and enhanced spatial memory functions. Interestingly, prolonged administration of metformin fails to achieve recovery from hyperglycemia, a key feature of diabetes, which may have a negative effect on hippocampal neurogenesis when it improves hippocampal abnormalities in middle‐aged DIO mice. Furthermore, metformin‐treated DIO mice display increased levels of phosphorylation of AMPK and atypical protein kinase C ζ (aPKC ζ), a downstream factor of AMPK 23, 24 in the hippocampus. In addition, the activation of selective phosphorylation sites of serine on hippocampal insulin receptor substrate 1 (IRS1), a member of the IRS family that are major mediators of the insulin/insulin‐like growth factor 1‐R (IGF1‐R) signaling pathway that is associated with the AMPK–aPKC ζ pathway 25, 26, 27, 28, is also observed corresponding to chronic therapy with metformin. These results suggest that chronic metformin‐stimulated serine phosphorylation of hippocampal IRS1 is involved in regulating adult neurogenesis and memory functions via interactions with AMPK/aPKC ζ signaling in a hypoglycemic effect‐independent manner.

## Materials and methods

### Animals

C57BL/6J male mice supplied by Japan SLC, Inc. (Shizuoka, Japan), were maintained at room temperature (25 ± 2 °C) under a standard 12‐h/12‐h light–dark cycle with free access to water and food. To determine the basic characteristics of DIO mice, C57BL/6J mice were fed an HFD (D12492, 60% kcal from fat; Research Diets, Inc., New Brunswick, NJ, USA) from 4 to 29 weeks of age while age‐matched control wild‐type (WT) mice were fed a normal diet (CE‐2; CLEA Japan, Tokyo, Japan). In the studies using middle‐aged DIO mice with metformin treatment (metformin‐treated DIO mice), for immunohistochemical analysis and behavioral testing, C57BL/6J mice received the HFD from 4 to 22 weeks of age, followed by the HFD plus chronic treatment with metformin (250 mg·kg^−1^·day^−1^) up to 35 weeks of age. For western blotting analysis, C57BL/6J mice were given the HFD from 4 to 34 weeks of age, followed by the HFD plus chronic treatment with metformin up to 45 weeks of age. Compared to metformin‐treated DIO mice, age‐matched (middle‐aged) DIO mice were fed the HFD only. Metformin treatments of WT mice were carried out from 8 or 32 weeks (8 months) of age for 3 weeks, with the metformin being administered in drinking water. To conduct the analysis between young and middle‐aged WT mice, C57BL/6J mice were fed a normal diet from 4 weeks of age up to 29 weeks of age. Behavioral and metabolic analyses were performed at 8 weeks of age (young WT mice) and 22–29 weeks of age (middle‐aged WT mice). Young WT mice with/without metformin treatment were sacrificed at 10 or 11 weeks of age for each analysis. Middle‐aged WT mice, middle‐aged WT mice with metformin treatment, middle‐aged DIO mice, and middle‐aged DIO mice with metformin treatment were sacrificed at 21–45 weeks of age. All mice were fasted for 6 h and anesthetized before sacrifice. All animal experiments were performed in compliance with the guidelines following approval by the Animal Care and Use Committee of Miyazaki University and National Center for Geriatrics and Gerontology (Obu, Japan).

### BrdU pulse‐chase labeling

Mice received intraperitoneal (IP) injections of BrdU (50 mg·kg^−1^ body weight) three times a day at 3‐h intervals. The mice were sacrificed at 3 h after the last injection on day 1 to examine hippocampal cell proliferation or at 10 days after the last injection to assess hippocampal cell differentiation. Brains were harvested from the mice and processed for immunohistochemistry.

### Immunohistochemistry

Whole brains were fixed in 4% paraformaldehyde overnight. Then, the fixative solution was changed to a 20% sucrose solution for dehydration. Brains were then sectioned at 25 or 50 μm thickness with a microtome. Free‐floating sections were permeabilized and blocked with 0.3% Triton X‐100 and 1% donkey serum in PBS. For BrdU immunostaining, sections were treated with 2 N HCl for 30 min at room temperature before blocking. Sections were incubated with primary antibodies overnight and then incubated with Alexa Fluor‐conjugated secondary antibodies (Molecular Probes, Waltham, MA, USA) for 1 h at room temperature to visualize primary antibody staining. Primary antibodies were rat anti‐BrdU (1 : 200; Abcam, Cambridge, MA, USA), rabbit anti‐Ki67 (1 : 500; Abcam), goat antidoublecortin (Dcx; 1 : 200; Santa Cruz Biotechnology, Santa Cruz, CA, USA), rabbit anti‐S100β (1 : 100; Abcam), rabbit anti‐Iba1 (1 : 500; Wako, Osaka, Japan), and mouse anti‐NeuN (1 : 100; Chemicon, Temecula, CA, USA). Confocal fluorescence images were obtained using confocal microscopes (A1, Nikon, Tokyo, Japan; and LSM710; Zeiss, Oberkochen, Germany).

### Quantification

The numbers of BrdU^+^, Ki67^+^, Dcx^+^, S100β^+^, Iba1^+^, and NeuN^+^ cells were counted under the confocal microscopes. For quantification, immunopositive cells were counted and the DG volume [granule cell layer (GCL), subgranular zone, and hilus except the molecular layer] was measured. Specifically, the DG volume was measured using a computerized setup for stereology driven by NIS‐Elements C/NIS‐Elements C‐ER (Nikon). The total number of immunopositive cells was normalized to the DG volume for presentation as the number of immunopositive cells per μm^3^. The GCL volume was measured per section (25 μm thickness) by immunostaining with anti‐NeuN antibody and calculated using NIS‐Elements C/NIS‐Elements C‐ER. To estimate the number of activated microglial cells, the size of the microglial cell body was measured (Nikon CL‐Quant) and activated microglia were defined by a cell body radius of > 3.6 μm. To analyze neuronal progenitor cell morphology, sections were stained with the anti‐Dcx antibody. Confocal Z stacks were obtained by Nikon A1. The total number of primary branches originating directly from the soma of Dcx‐positive cells was manually counted through the hippocampus.

### Water T maze

Hippocampal‐dependent spatial memory was tested using a water T maze 29, 30. The maze consisted of a start box, left arm, and right arm, which was filled with water at 23 °C (± 1 °C) up to 1 cm above the surface of the platform. Mice were placed in the start box and allowed to swim to the right or left arm and then placed back in the home cage. This screening step was repeated three times at 15‐s intervals. The platform was placed on the side that mice reached less often. Next, mice were allowed to explore the maze freely. If mice reached the platform, they were allowed to rest there for 5 s (correct choice). If not, the arm entry was closed with a board and they were forced to swim for 15 s as a deterrent (incorrect choice). This trial step was repeated five times at 4‐min intervals. Mice were subjected to this trial step for 5 days. For the evaluation of the results of this trial, the percentage of correct responses per day was calculated.

### Measurement of metabolic parameters

Body weight was recorded weekly throughout the study. The amount of food intake in a day was recorded after 24‐h fasting. The level of blood glucose at 6‐h fasting was measured using a portable glucose meter (ACCU‐CHEK^®^ Aviva; Roche DC Japan K.K., Tokyo, Japan). The level of plasma insulin at 6‐h fasting was determined using an insulin enzyme‐linked immunosorbent assay kit (Morinaga, Yokohama, Japan). The levels of plasma free fatty acid (FFA), triglycerol (TG), total cholesterol (T‐CHO), low‐density lipoprotein (LDL) cholesterol (LDL‐C), and high‐density lipoprotein (HDL)‐cholesterol (HDL‐C) at 6‐h fasting were assayed using enzymatic methods (Oriental Yeast Co., Ltd., Tokyo, Japan).

### Western blotting

Hippocampal tissue was isolated on ice and homogenized in lysis buffer [T‐PER tissue protein extract reagent (Thermo Scientific, Waltham, MA, USA) containing protease inhibitor cocktail (Thermo Scientific) and phosphatase inhibitor cocktail (Nacalai Tesque, Kyoto, Japan)] with a pellet mixer. After incubation on ice for 15 min, the lysates were centrifuged for 5 min at 14 200 ***g*** and 4 °C. The supernatants were placed in a fresh tube. Protein concentration was calculated using BCA protein assay kit (Pierce, Rockford, IL, USA). The aliquot of hippocampal lysates was boiled for 5 min in Laemmli SDS sample buffer [60 mm Tris/Cl (pH 6.8), 2% sodium dodecyl sulfate, 10% glycerol, 4% β‐mercaptoethanol, and 0.01% bromophenol blue]. A total of 15 μg of each SDS protein sample was loaded per lane, separated by 7.5% SDS/PAGE, and transferred to nitrocellulose membranes. Membranes were blocked using 4% block ace (Yukijirushi Co., Tokyo, Japan) at room temperature for 1 h, incubated with the indicated primary antibodies at 4 °C overnight, followed by incubation with horseradish peroxidase‐conjugated secondary antibodies at room temperature for 2 h. Primary antibodies were rabbit anti‐phospho‐IR β (Tyr1135/Tyr1136; 1 : 1000; Cell Signaling Technology, Danvers, MA, USA), rabbit anti‐IR β (1 : 1000; Cell Signaling Technology), rabbit anti‐phospho‐IRS1 [mouse Ser307/human Ser312 (mSer307/hSer312), mouse Ser612/human Ser616 (mSer612/hSer616), mouse Ser632/Ser635/human Ser636/Ser639 (mSer632/Ser635/hSer636/Ser639), mouse Ser1097/human Ser1101 (mSer1097/hSer1101)] (1 : 500; Cell Signaling Technology), rabbit anti‐IRS1 (1 : 1000; Cell Signaling Technology), rabbit anti‐phospho‐AMPK (Thr172; 1 : 1000; Cell Signaling Technology), rabbit anti‐AMPK (1 : 1000; Cell Signaling Technology), rabbit anti‐phospho‐aPKC ζ/λ (Thr410/Thr403; 1 : 500; Cell Signaling Technology), rabbit anti‐aPKC λ/ι (1 : 500; Santa Cruz Biotechnology), rabbit anti‐phospho‐mTOR (Ser2448; 1 : 1000; Cell Signaling Technology), rabbit anti‐mTOR (1 : 1000; Cell Signaling Technology), and rabbit anti‐β‐tubulin (1 : 1000; Cell Signaling Technology). The chemiluminescent signals were detected using Chemi‐Lumi One (Nacalai Tesque) or ImmunoStar LD (Wako). The images were scanned with a LAS‐4000 (GE Healthcare, Chicago, IL, USA) instrument.

### Statistics

All results are presented as mean ± standard error of the mean (SEM). Data were statistically analyzed using Student's *t*‐test. Significance is indicated as *P < 0.05 and **P < 0.01.

## Results

### Adult neurogenesis and memory function are impaired in the hippocampus of middle‐aged DIO mice

**Figure 1:**
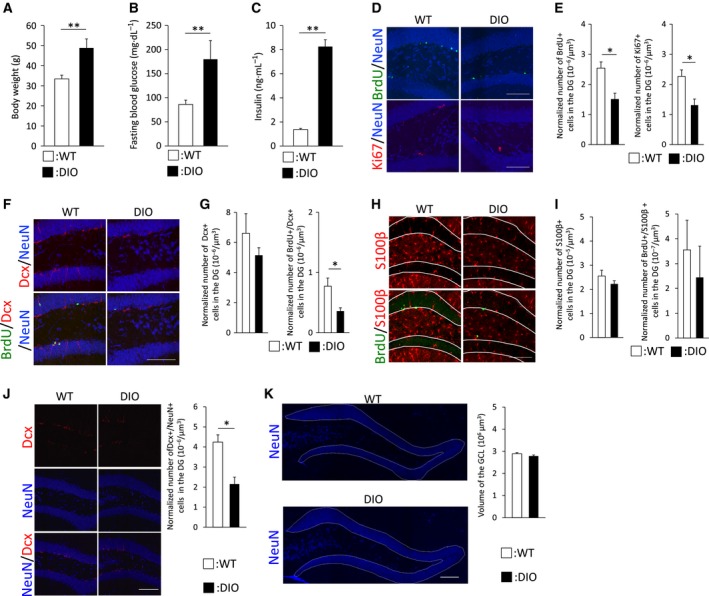
Middle‐aged DIO mice have decreased hippocampal neurogenesis and impaired spatial memory functions. (A–C) Metabolic parameters of middle‐aged WT mice or DIO mice. Representative graphs display body weights (middle‐aged WT and DIO mice, *n* = 15 animals; 25 weeks of age) (A), 6‐h fasting blood glucose levels (middle‐aged WT and DIO mice, *n* = 12 animals; 25 weeks of age) (B), or 6‐h fasting blood insulin levels (middle‐aged WT mice and DIO mice, *n* = 14 animals; 22–24 weeks of age) (C). (D) Immunohistological analysis of cell proliferation. Representative images show BrdU (green), Ki67 (red), or NeuN (blue) staining in DG of middle‐aged WT mice (*n* = 3 animals; 21 weeks of age) or DIO mice (*n* = 3 animals; 21 weeks of age). (E) Graphs show quantification of BrdU^+^ or Ki67^+^ cells in DG of middle‐aged WT or DIO mice. (F,H) Tracing cell differentiation by immunostaining. Representative images display BrdU (green), Dcx (red), NeuN (blue) (F), BrdU (green), and S100β (red) (H) staining in DG of middle‐aged WT (*n* = 3 animals; 24 weeks of age) or DIO mice (*n* = 3 animals; 24 weeks of age). White lines denote the GCL. (G,I) Graphs present quantitative comparisons of Dcx^+^, BrdU^+^/Dcx^+^ (G), S100β^+^, and BrdU^+^/S100β^+^ cells (I) in DG of middle‐aged WT or DIO mice. (J) Analysis of newly generated mature neurons in DG. Representative images show Dcx (red) and NeuN (blue) staining in DG of middle‐aged WT (*n* = 3 animals; 21 weeks of age) or DIO mice (*n* = 3 animals; 21 weeks of age). Graphs present quantitative analysis of NeuN^+^/Dcx^+^ cells in DG of middle‐aged WT or DIO mice. (K) Visualization of GCL by staining with an antibody against NeuN. The region of interest (ROI) is indicated by a white line. Graphs show quantification of the estimated GCL volume per section in middle‐aged WT (*n* = 3 animals; 21 weeks of age) or DIO mice (*n* = 3 animals; 21 weeks of age). Data are mean ± SEM. Significances were determined using Student's *t*‐test (*P < 0.05; **P < 0.01).

Hippocampal neurogenesis is implicated in hippocampal‐dependent learning and memory functions. Previous studies have shown that aging and diabetes decrease hippocampal neurogenesis, which involves cognitive impairment in the hippocampus 3, 4, 5. However, it is unclear to what degree the combination of aging and diabetes affects neurogenesis and memory functions in the hippocampus. Therefore, we investigated hippocampal neurogenesis in middle‐aged DIO mice, a physiological type 2 diabetic mouse model. Consistent with the findings in previous studies, middle‐aged DIO mice manifested obesity, hyperglycemia, and hyperinsulinemia under our experimental conditions (Fig. 1A–C). First, we evaluated cell proliferation in the hippocampal DG of middle‐aged DIO mice. Immunostaining with antibodies against BrdU, which is incorporated into DNA during S phase of the cell cycle, or Ki67, a nuclear antigen expressed during the cell cycle, revealed that the number of BrdU‐labeled or Ki67‐positive cells was profoundly decreased in middle‐aged DIO mice compared with that in age‐matched WT mice (Fig. 1D,E). Next, we examined the impact of HFD‐induced diabetes on cell differentiation in DG of the hippocampus in those mice (Fig. 1F,H). The number of BrdU‐retaining and Dcx (immature neuron marker)‐expressing new neurons was remarkably decreased in middle‐aged DIO mice compared with that in age‐matched WT mice, whereas no significant difference in the total number of Dcx‐positive immature neurons was observed between these two groups (Fig. 1G). Meanwhile, the numbers of newly generated BrdU and S100β (astrocyte marker) double‐positive astrocytes and S100β‐positive astrocytes were comparable (Fig. 1I). These results raised an additional issue regarding the effect of the decrease in new neurons on NeuN‐positive cells and the GCL volume in DG of middle‐aged DIO mice. Consequently, immunostaining for Dcx and NeuN, a neuronal nuclear antigen expressed in mature neurons, revealed that the number of Dcx/NeuN double‐positive neurons was notably decreased in middle‐aged DIO mice (Fig. 1J), whereas there was no significant difference in the GCL volume between the two groups (Fig. 1K). These results suggest that type 2 diabetes negatively influences the production of new neurons in DG independent of volume regulation of the GCL, which is consistent with the findings of a previous study 31. Thus, HFD‐induced type 2 diabetes dramatically decreases hippocampal neurogenesis in middle‐aged mice.

### Middle‐aged DIO mice display hippocampus‐dependent cognitive decline

**Figure 2:**
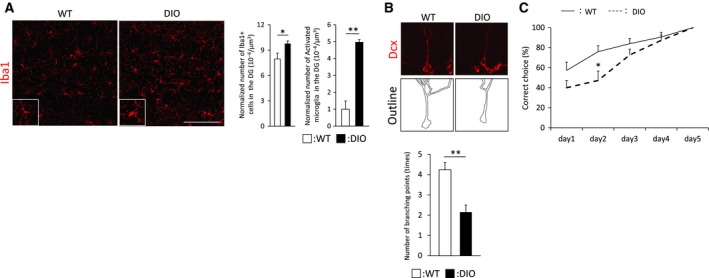
Hippocampus‐dependent spatial memory is impaired in middle‐aged DIO mice. (A) Immunohistological analysis of microglia. Representative images show Iba1 (red) staining in DG of middle‐aged WT (*n* = 3 animals; 21 weeks of age) or DIO mice (*n* = 3 animals; 21 weeks of age). High‐magnification images of activated microglia are shown at the lower left. Graphs show quantitative comparisons of Iba1^+^ cells and activated microglia expressing Iba1 in DG of middle‐aged WT or DIO mice. Middle‐aged DIO mice exhibit impairment in spatial memory functions. (B) High‐magnification images obtained by immunostaining with an anti‐Dcx antibody. Graphs indicate the number of dendritic branching points of Dcx^+^ cells in middle‐aged WT (*n* = 3 animals; 21 weeks of age) or DIO mice (*n* = 3 animals; 21 weeks of age). Scale bar: 20 μm. (C) Evaluation of learning memory functions in middle‐aged WT (*n* = 15 animals; 22–29 weeks of age) or DIO mice (*n* = 15 animals; 22–29 weeks of age) by a water T maze. Data are mean ± SEM. Significances were determined using Student's *t*‐test (*P < 0.05; **P < 0.01).

Brain inflammation is associated with deficits in adult neurogenesis and functions in the hippocampus 32, 33, 34. To investigate the effect of the combination of aging and diabetes on microglial activity in the hippocampal DG, we performed immunohistochemical analysis with an antibody against Iba1 (a microglia marker). Activated microglia were defined by a cell body radius of > 3.6 μm. The number of Iba1‐positive cells and activated microglia expressing Iba1 was profoundly increased in DG of middle‐aged DIO mice (Fig. 2A). Furthermore, we examined the dendritic integrity of neuroblasts, which is related to spatial memory performance and decreases with aging or diabetes in middle‐aged DIO mice 35, 36, 37. Morphological analysis of Dcx‐positive cells revealed a reduction in the complexity of dendritic arborizations in DG of those mice, as indicated by the decreased number of branch points (Fig. 2B).

Altogether, these results indicate that the combination of aging and type 2 diabetes causes a prominent reduction in adult neurogenesis accompanied by increased microglial activation and impaired dendritic complexity in the hippocampal DG.

Subsequently, we assessed whether the significant reduction in dendritic integrity affects spatial memory in middle‐aged DIO mice. The water T maze test, an improved hippocampus‐dependent learning and memory task 29, 30, showed that the percentage of correct choices of middle‐aged DIO mice was lower than that of age‐matched WT mice, whereas no significant difference was observed in the number of days required to achieve 100% accuracy between the two groups (Fig. 2C). Notably, on day 2, middle‐aged DIO mice had significantly lower percentages of correct choices compared with age‐matched WT mice (Fig. 2C). Our results indicated that HFD‐induced type 2 diabetes further deteriorates hippocampus‐dependent memory functions with aging, which is consistent with previous studies showing that young adult animal models of diabetes exhibit cognitive impairment 4, 5.

### Chronic treatment with metformin restores hippocampal neurogenesis in middle‐aged DIO mice

In addition to metformin‐induced antidiabetic effects, metformin treatment enhances hippocampal neurogenesis and cognitive functions in cancer drug‐ and neurotoxin‐induced neurodegenerative animal models 21, 22. However, it is unclear whether chronic metformin treatment influences hippocampal neurogenesis in young and middle‐aged WT mice receiving regular chow and middle‐aged WT mice receiving HFD (DIO mice). Basically, hippocampal‐dependent memory function and the levels of fasting blood glucose were comparable between young and middle‐aged WT mice (Fig. S1A,C), whereas aging robustly increased body weight in WT mice (Fig. S1B). First, we examined the effect of chronic metformin treatment on hippocampal neurogenesis in young and middle‐aged WT mice receiving regular chow, to obtain basic data. A previous study showed that IP or intravenous (IV) injection of metformin (no detailed description of the injection method is available) for 12 days increased cell proliferation and neuronal differentiation in 8‐week‐ or 7‐month‐old WT mice receiving regular chow (no detailed description of the gender is available) compared with those mice without metformin injection 24. By contrast, chronic oral metformin administration in drinking water for 3 weeks had no effect on cell proliferation and neuronal differentiation in 8‐week‐ or 8‐month‐old male WT mice receiving regular chow (Fig. S2A–H). Meanwhile, there were no differences in body weight and fasting blood glucose between young or middle‐aged WT mice and young or middle‐aged WT mice with chronic metformin treatment (Fig. S2I,J). Our data indicate that, in young and middle‐aged WT mice, long‐term metformin administration in drinking water has no effect on blood glucose levels and hippocampal neurogenesis, suggesting that chronic oral metformin administration may be insufficient for altering hippocampal neurogenesis.

**Figure 3:**
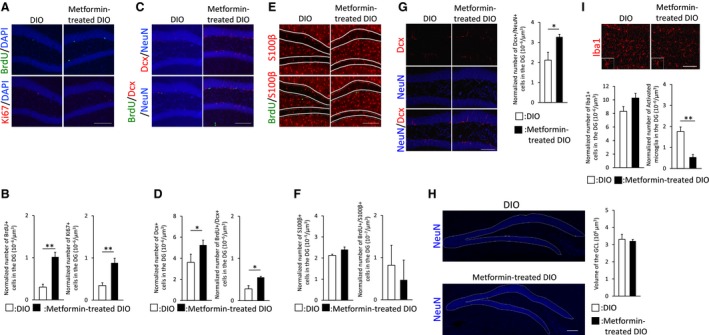
Middle‐aged DIO mice with chronic metformin treatment display enhanced hippocampal neurogenesis. (A) Immunohistological analysis of cell proliferation markers. Representative images show BrdU (green), Ki67 (red), or DAPI (blue) staining in DG of middle‐aged DIO (*n* = 4 animals; 35 weeks of age) or metformin‐treated DIO mice (*n* = 4 animals; 35 weeks of age). (B) Graphs show quantitative analysis of BrdU^+^ cells or Ki67^+^ cells in DG of middle‐aged DIO or metformin‐treated DIO mice. (C,E) Tracking cell differentiation by immunostaining. Representative images display BrdU (green), Dcx (red), NeuN (blue) (C), BrdU (green), and S100β (red) (E) staining in DG of middle‐aged DIO (*n* = 4 animals; 35 weeks of age) or metformin‐treated DIO mice (*n* = 4 animals; 35 weeks of age). (D,F) Graphs present quantification of Dcx^+^, BrdU^+^/Dcx^+^ (D), S100β^+^, and BrdU^+^/S100β^+^ cells (F) in DG of middle‐aged DIO or metformin‐treated DIO mice. (G) Analysis of new mature neurons in DG. Representative images of DG stained for NeuN (blue) and Dcx (red). Graphs show quantitative comparison of NeuN^+^/Dcx^+^ cells in DG of middle‐aged DIO (*n* = 4 animals; 35 weeks of age) or metformin‐treated DIO mice (*n* = 4 animals; 35 weeks of age). (H) GCL is shown by the depicted ROI in middle‐aged DIO (*n* = 4 animals; 35 weeks of age) or metformin‐treated DIO mice (*n* = 4 animals; 35 weeks of age). Graphs show quantitative comparison of the estimated GCL volume per section in middle‐aged DIO or metformin‐treated DIO mice. (I) Analysis of microglial activation. Representative images show Iba1 (red) staining in DG of middle‐aged DIO (*n* = 4 animals; 35 weeks of age) or metformin‐treated DIO mice (*n* = 4 animals; 35 weeks of age). High‐magnification images at the lower left display activated microglia. Graphs show quantitative analysis of Iba1^+^ cells and activated microglia in DG of middle‐aged DIO and metformin‐treated DIO mice. Scale bar: 100 μm. Data are the mean ± SEM. Significances were determined using Student's *t*‐test (*P < 0.05; **P < 0.01).

Next, we investigated whether chronic oral metformin treatment influences the impairment of hippocampal neurogenesis and cognitive performance in middle‐aged DIO mice. Immunohistochemical analyses of cell proliferation using antibodies against BrdU or Ki67 revealed that long‐term treatment with metformin dramatically increased the number of cells labeled with BrdU or expressing Ki67 in the hippocampal DG of middle‐aged DIO mice (Fig. 3A,B). We also performed double immunostaining for BrdU and cell‐specific markers including Dcx or S100β to assess cell differentiation (Fig. 3C,E). The number of Dcx single‐positive immature neurons and the number of new Dcx‐positive immature neurons retaining BrdU were evidently elevated in DG of metformin‐treated DIO mice (Fig. 3D). By contrast, there were no significant differences in the number of S100β single‐positive astrocytes and the number of newly generated S100β‐positive astrocytes retaining BrdU between metformin‐treated DIO and middle‐aged DIO mice (Fig. 3F). Moreover, we examined the effect of prolonged treatment with metformin on newly generated mature neurons and the GCL volume. Double immunostaining using antibodies against NeuN and Dcx showed that metformin‐treated DIO mice displayed an increased number of Dcx/NeuN double‐positive cells in the hippocampal DG compared with DIO mice (Fig. 3G), whereas the GCL volume did not differ between the two groups (Fig. 3H), indicating that chronic therapy with metformin facilitates the generation of neurons without altering the GCL volume. The mechanism responsible for the disparity between the generation of neurons and the integration of these neurons is unknown. However, it has been suggested that functions mediated through niche signals are required for the integration of new neurons 35, 38, 39, 40. Thus, long‐term metformin treatment has a positive effect on cell proliferation and neuronal differentiation in middle‐aged DIO mice.

As shown in Fig. 2A, middle‐aged DIO mice exhibited an increase in microglial activation. We further investigated whether prolonged therapy with metformin influences chronic microglial activation in DG of middle‐aged DIO mice.

Although the total number of Iba1‐positive cells was comparable between metformin‐treated DIO and DIO mice, chronic metformin administration dramatically reduced the number of activated microglia in DIO mice (Fig. 3I). Taken together, our data demonstrate that long‐term therapy with metformin enhances hippocampal neurogenesis in middle‐aged DIO mice while inhibiting chronic microglial activation.

### Long‐term metformin treatment restores memory performance in middle‐aged DIO mice without improving glucose metabolism

**Figure 4:**
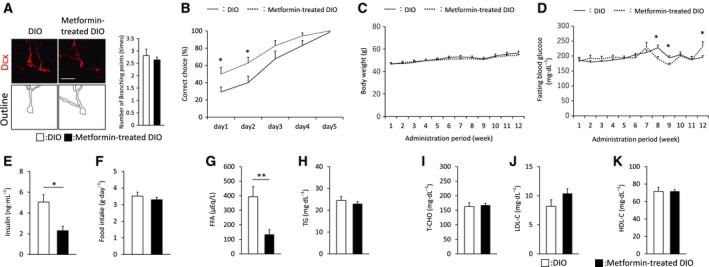
Chronic metformin treatment has a positive effect on memory functions in a hypoglycemic effect‐independent manner. (A) Brain sections were stained with an anti‐Dcx antibody. High‐magnification images were obtained by confocal microscopy. Graphs show quantification of dendritic branching points of Dcx^+^ cells in middle‐aged DIO (*n* = 4 animals; 35 weeks of age) or metformin‐treated DIO mice (*n* = 4 animals; 35 weeks of age). Scale bar: 20 μm. (B) Evaluation of the spatial memory of middle‐aged DIO (*n* = 13 animals; 29–35 weeks of age) or metformin‐treated DIO mice (*n* = 14 animals; 29–35 weeks of age) by the water T maze. (C,D) The time course for changes in body weights (C) or 6‐h fasting blood glucose levels (D) of middle‐aged DIO (*n* = 8 animals; 22–34 weeks of age) or metformin‐treated DIO mice (*n* = 8 animals; 22–34 weeks of age). (E) Quantitative comparison of 6‐h fasting blood insulin levels of middle‐aged DIO (*n* = 8 animals; 34 weeks of age) or metformin‐treated DIO mice (*n* = 8 animals; 34 weeks of age). (F) The graph shows food intake of middle‐aged DIO (*n* = 4 animals; 31 weeks of age) or metformin‐treated DIO mice (*n* = 4 animals; 31 weeks of age). (G–K) Biochemical parameters including FFA (G), TG (H), T‐CHO (I), LDL‐C (J), and HDL‐C (K) were analyzed in 6‐h fasting plasma of DIO (*n* = 8 animals; 34 weeks of age) or metformin‐treated DIO mice (*n* = 8 animals; 34 weeks of age). Data are the mean ± SEM. Significances were determined using Student's *t*‐test. *P < 0.05; **P < 0.01.

Next, the impact of chronic metformin treatment on dendritic complexity was investigated in DG of middle‐aged DIO mice. Immunostaining with anti‐Dcx antibody showed that prolonged treatment with metformin has no effect on the number of dendritic branching points of Dcx‐positive cells (Fig. 4A). Subsequently, we evaluated whether chronic therapy with metformin affects spatial memory functions in middle‐aged DIO mice. Behavioral tests showed that metformin‐treated DIO mice made significantly greater percentages of correct choices than middle‐aged DIO mice on days 1 and 2, whereas long‐term metformin treatment was ineffective in changing the number of days to reach 100% accuracy (Fig. 4B). These results indicate that long‐term metformin treatment ameliorates cognitive decline in middle‐aged DIO mice without influencing dendritic integrity, suggesting a beneficial effect of prolonged metformin treatment on memory functions, which is consistent with the findings of previous studies 21, 22, 35, 38, 39, 40.

It was important to investigate whether metformin‐induced improvement of hippocampal deterioration in middle‐aged DIO mice is involved in recovery from type 2 diabetes. Previous studies have shown that long‐term metformin treatment has no effect on body weight or blood glucose levels in HFD‐fed mice 41, 42. Consistent with these results, prolonged treatment with metformin failed to improve weight gain, elevated levels of fasting blood glucose, and feeding behavior in middle‐aged DIO mice (Fig. 4C,D,F), whereas blood insulin level was significantly decreased in metformin‐treated DIO mice (Fig. 4E). The conflicting results on the effects of metformin on blood glucose between animals and humans may be due to differences in species‐specific effects of metformin. We also evaluated the effect of chronic metformin treatment on diabetes‐associated alterations in lipid metabolism in middle‐aged DIO mice. Hippocampal cell proliferation has been reported to be reduced in young WT mice fed normal diets with an increased level of FFA or HFD (45% calories from fat) with FFA 43. Correspondingly, metformin‐treated DIO mice exhibited a decreased plasma level of FFA compared with DIO mice without metformin administration (Fig. 4G), although the plasma levels of TG, T‐CHO, LDL, and HDL were unchanged between the two groups (Fig. 4H–K). Altogether, our results show that chronic therapy with metformin restores hippocampal neurogenesis and hippocampus‐dependent memory functions accompanied by reduced plasma levels of insulin and FFA in middle‐aged DIO mice, independently of the hypoglycemic effect.

### Chronic metformin treatment increases the phosphorylation of hippocampal AMPK/aPKC ζ/IRS1

**Figure 5:**
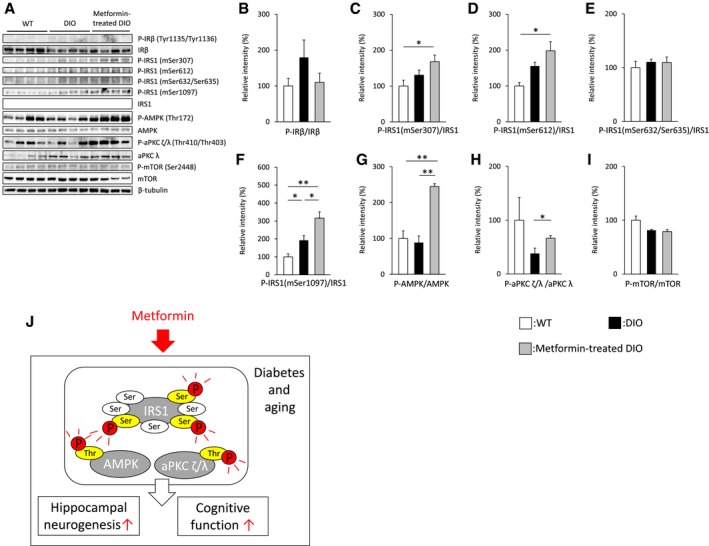
Long‐term metformin treatment stimulates the phosphorylation of IRS1 at some serine residues. (A) Total and phosphorylated forms of hippocampal proteins isolated from middle‐aged WT (*n* = 4 animals; 35 weeks of age), middle‐aged DIO (*n* = 4 animals; 45 weeks of age), or metformin‐treated DIO mice (*n* = 4 animals; 45 weeks of age) were detected by specific antibodies. Levels of phosphorylation were normalized by levels of total protein expression. β‐Tubulin was used as a loading control. (B–I) Graphs present relative levels of phosphorylated insulin receptor β [B], IRS1 (mSer307, mSer612, mSer632/Ser635, or mSer1097) [C–F], AMPK [G], aPKC ζ/λ [H], and mTOR [I]. (J) Schematic representation of metformin‐stimulated phosphorylation of AMPK/IRS1/ aPKC ζ/λ. Data are the mean ± SEM. Significances were determined using Student's *t*‐test. *P < 0.05; **P < 0.01.

To gain insight into the mechanism behind the improvement in adult neurogenesis and spatial memory in metformin‐treated DIO mice with reduced plasma levels of insulin and FFA, we examined the levels of protein and phosphorylation of metformin‐related signaling pathways such as the AMPK–mTOR/aPKC ζ/λ pathway and IR–IRS1 signaling in the hippocampus of middle‐aged DIO mice with or without chronic treatment with metformin 23, 24, 44, 45, 46, 47, 48. IP injection of metformin for 1 or 14 days increased the phosphorylation level of AMPK in the hippocampus or the whole brain of disease model animals 45, 49. Similarly, in our studies, chronic metformin administration in drinking water augmented the phosphorylation level of AMPK in middle‐aged DIO mice, whereas the phosphorylation level of mTOR was unchanged in those mice (Fig. 5A,G,I). Consistent with the findings of previous studies showing that metformin stimulates aPKC ζ/λ activity in culture cells 23, 24, the phosphorylation level of aPKC ζ/λ was significantly increased in metformin‐treated DIO mice (Fig. 5A,H).

A high plasma FFA level in adipose cells induces phosphorylation at the Ser307 site in IRS1, which is decreased in skeletal muscle of obese animals with chronic oral metformin treatment 50, 51. However, contrary to the findings of *in vitro* experiments, IRS1 mSer307 knock‐in mice demonstrated that IRS1 mSer307 is a positive regulatory site that is essential for normal insulin signaling 52. Corresponding with this, metformin‐treated DIO mice displayed a significant increase in the phosphorylation level of IRS1 mSer307 in the hippocampus, independently of the plasma levels of insulin, glucose, and FFA (Fig. 5A,C), although the phosphorylation levels of hippocampal IR β‐subunits were comparable between DIO mice and metformin‐treated DIO mice (Fig. 5A,B). On the other hand, both mSer612 and mSer632/635 sites in IRS1 negatively correlate with the tyrosine phosphorylation of IRS1, which provokes insulin signaling 53. Similarly, the phosphorylation level of hippocampal IRS1 mSer612 was significantly elevated in metformin‐treated DIO mice regardless of the plasma concentrations of insulin, glucose, and FFA, whereas the phosphorylation levels of mSer632/635 on IRS1 were unchanged in the hippocampus between the groups (Fig. 5A,D,E). Meanwhile, chronic metformin treatment further promoted the phosphorylation level of IRS1 mSer1097, a target of mTOR signaling in the liver, in obese model animals 54, which was also augmented in the hippocampus of middle‐aged DIO mice with hyperglycemia compared with that in age‐matched WT mice, regardless of the level of mTOR phosphorylation (Fig. 5A,F,I). Taken together, our findings suggest that chronic metformin‐stimulated phosphorylation of AMPK/aPKC ζ/λ/IRS1 serine sites in the hippocampus is involved in neuroprotective effects on adult neurogenesis and memory function.

## Discussion

Our study demonstrates that long‐term therapy with oral metformin enhances hippocampal neurogenesis and spatial memory accompanied by the inhibition of chronic microglial activation and increased phosphorylation of AMPK/aPKC ζ/λ/IRS1 serine residues in the hippocampus of middle‐aged diabetic mice independently of a glucose‐lowering effect. These results are consistent with previous studies describing that chronic oral metformin administration has neuroprotective effects on HFD‐induced decline in hippocampal neurogenesis and neurological disorders including dementia in animals and humans 10, 12, 14, 15, 55, 56, 57, 58. Similarly, in animal disease models, chronic or acute IP injection of metformin improves neurodegeneration, cognitive decline, and neuroinflammation 45, 49, 57. These findings suggest that metformin exerts direct beneficial effects on brain functions, although its hypoglycemic effect is predominantly mediated via the gut 15.

Furthermore, Wang *et al*. 24 showed that IP or IV injection of metformin (no detailed description of the injection method is available) for 12 days enhanced hippocampal neurogenesis in both 8‐week‐ and 7‐month‐old WT mice receiving regular chow. Nonetheless, in our study, chronic oral metformin administration in drinking water had no effect on adult hippocampal neurogenesis in both 8‐week‐ and 8‐month‐old WT mice receiving regular chow (Fig. S2A–H). On the other hand, chronic metformin treatment failed to restore cognitive decline in old mice or rat models of HFD‐induced obesity 59, 60 and moreover may also be related to an increased risk of AD in animals or the elderly 13, 61. These contradictory assertions may be due to differences in the metformin treatment protocols, such as age of exposure, drug dose, duration of treatment, administration method, genetic background, and severity of disease. Thus, the effects of metformin treatment on brain functions, including hippocampal neurogenesis and memory functions, over species remain unclear.

The insulin/IGF‐1 signaling pathway plays a pivotal role in the regulation of glucose metabolism, aging, and longevity 62. IGF1‐R → IRS2 signaling in the brain modulates life span and its reduction inhibits symptoms in AD or HD model mice; however, suppression of IR in the brain fails to rescue these symptoms, suggesting distinct functions of IGF1‐R → IRS2 signaling and IR signaling in the brain 63, 64, 65, 66, 67, 68, 69. IGF1 signaling in neural stem cells negatively regulates olfactory bulb neurogenesis and olfactory sensory function 70, and neural IRS2 also appears to be involved in modulating adult neurogenesis and memory function (Taguchi A, unpublished data). However, the role of insulin in regulating adult neurogenesis still remains unknown because both animal models of type 2 diabetes with insulin resistance and insulin‐deficient type 1 diabetes exhibit reduced hippocampal neurogenesis and impaired memory performance 71, 72, 73, 74, 75. Therefore, it is unknown whether a decreased level of plasma insulin contributes to improvement in neurogenesis and spatial memory in the hippocampus of metformin‐treated DIO mice (Fig. 4E). On the other hand, recent studies showed that intranasal insulin reduces cognitive decline and pathology in AD model animals 76, 77. These data suggest controversial effect of insulin on brain functions. Indeed, the present study shows, when prolonged treatment with metformin rescues impaired neurogenesis and cognitive deficits in middle‐aged DIO mice, phosphorylation of AMPK/aPKC ζ/λ/IRS1 serine residues in the hippocampus of those mice occurs regardless of the plasma levels of insulin, FFA, and glucose (Fig. 5A,C,F–H).

Because mice with homozygous disruption of IRS1 display mild phenotypes such as growth retardation and mild insulin resistance, but not diabetes 78, 79, little is known about the function of IRS1 in the brain. The multiserine/threonine (S/T) phosphorylation sites on IRS1 positively and negatively regulate the signaling pathways in both insulin‐dependent and insulin‐independent manners, affecting phosphorylation and dephosphorylation of tyrosine and other S/T on IRS1 53, 80. However, the functions and the regulatory mechanisms of S/T phosphorylation sites of IRS1 remain unclear *in vivo* due to the large number of S/T residues found on IRS1 in various tissues/cells of humans and animals. Our findings indicate that long‐term therapy with metformin induces high levels of phosphorylated hippocampal IRS1 at three serine residues, namely mSer307, mSer612, and mSer1097, in DIO mice independently of a hypoglycemic effect when increased phosphorylation of AMPK and aPKC ζ/λ occurs in the hippocampus of metformin‐treated DIO mice (Fig. 5A,C,D,G,H). Of the three serine residues on IRS1, increased levels of phosphorylation at mSer307 (hSer312) and mSer612 (hSer616) are observed in AD hippocampus and cerebral cortex 81, 82, 83, whereas the phosphorylation level of mSer632/635 (hSer 636/639), which is also elevated in the brain from patients with AD or AD‐transgenic model mice, is comparable in the hippocampus between groups (Fig. 5A,E). These discrepancies are probably due to compensatory effects produced by metformin‐stimulated and/or context‐dependent phosphorylation of serine residues on IRS1, such as positive and negative feedback mechanisms. Although *in vitro* studies have shown that IRS1 is associated with the regulation of neural stem cell proliferation 84, it is difficult to elucidate the connection between hippocampal cell proliferation and IRS1 *in vivo* because there are currently few reliable IRS1 antibodies that are available commercially for immunohistochemical analyses. Nevertheless, whether IRS1 and its serine phosphorylation induced by chronic metformin treatment are involved in modulating hippocampal neurogenesis and memory functions *in vivo* needs to be addressed by future studies, including analysis of brain‐specific IRS1‐knockout mice.

In summary, we have shown that chronic oral metformin‐stimulated phosphorylation of selective serine residues on IRS1 is related to hypoglycemic action‐independent enhancements of neurogenesis and memory functions through increased phosphorylation of AMPK/aPKC ζ/λ in the hippocampus and may be a potential marker for the assessment of metformin‐induced alterations in the hippocampus.

## Author contributions

AT conceived and designed the project. DT, EK, WF, KK, MK, and HT acquired the data, and DT, EK, WF, KK, MK, and MN analyzed and interpreted the data. DT and AT wrote the manuscript.

## Supporting information


**Fig. S1**. Middle‐aged WT mice exhibit normal cognitive performance similar to young WT mice regardless of age.

**Fig. S2**. Chronic metformin treatment has no effect on hippocampal neurogenesis in young and middle‐aged WT mice.
