# Erratum to: Metformin treatment ameliorates diabetes‐associated decline in hippocampal neurogenesis and memory via phosphorylation of insulin receptor substrate 1

**DOI:** 10.1002/2211-5463.12536

**Published:** 2018-10-22

Daisuke Tanokashira, Eiko Kurata, Wataru Fukuokaya, Kenshiro Kawabe, Mana Kashiwada, Hideyuki Takeuchi, Masamitsu Nakazato and Akiko Taguchi

In the paper by Tanokashira *et al*. [1], there was an error in Fig. 1, part 1a. The correct figure is reproduced below.

**Figure 1:**
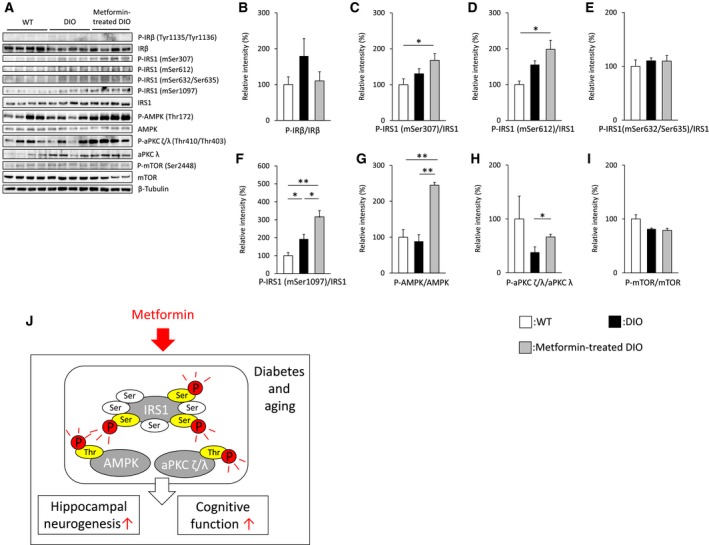
Long‐term metformin treatment stimulates the phosphorylation of IRS1 at some serine residues. (A) Total and phosphorylated forms of hippocampal proteins isolated from middle‐aged WT (*n* = 4 animals; 35 weeks of age), middle‐aged DIO (*n* = 4 animals; 45 weeks of age), or metformin‐treated DIO mice (*n* = 4 animals; 45 weeks of age) were detected by specific antibodies. Levels of phosphorylation were normalized by levels of total protein expression. β‐Tubulin was used as a loading control. (B–I) Graphs present relative levels of phosphorylated insulin receptor β [B], IRS1 (mSer307, mSer612, mSer632/Ser635, or mSer1097) [C–F], AMPK [G], aPKC ζ/λ [H], and mTOR [I]. (J) Schematic representation of metformin‐stimulated phosphorylation of AMPK/IRS1/ aPKC ζ/λ. Data are the mean ± SEM. Significances were determined using Student's *t*‐test. *P < 0.05; **P < 0.01.

**Reference**

1 Tanokashira D, Kurata E, Fukuokaya W, Kawabe K, Kashiwada M, Takeuchi H, Nakazato M and Taguchi A (2018) Metformin treatment ameliorates diabetes‐associated decline in hippocampal neurogenesis and memory via phosphorylation of insulin receptor substrate 1. *FEBS Open Bio* **8,** 1104–1118. https://doi.org/10.1002/2211-5463.12436

