# Supplementary figures and images for: Metformin treatment ameliorates diabetes‐associated decline in hippocampal neurogenesis and memory via phosphorylation of insulin receptor substrate 1
Source: FEBS Open Bio. 2018 May 18;8(7):1104–18. doi: 10.1002/2211-5463.12436 (PMC6026705; doi:10.1002/2211-5463.12436)

Supplemental Figure S1.

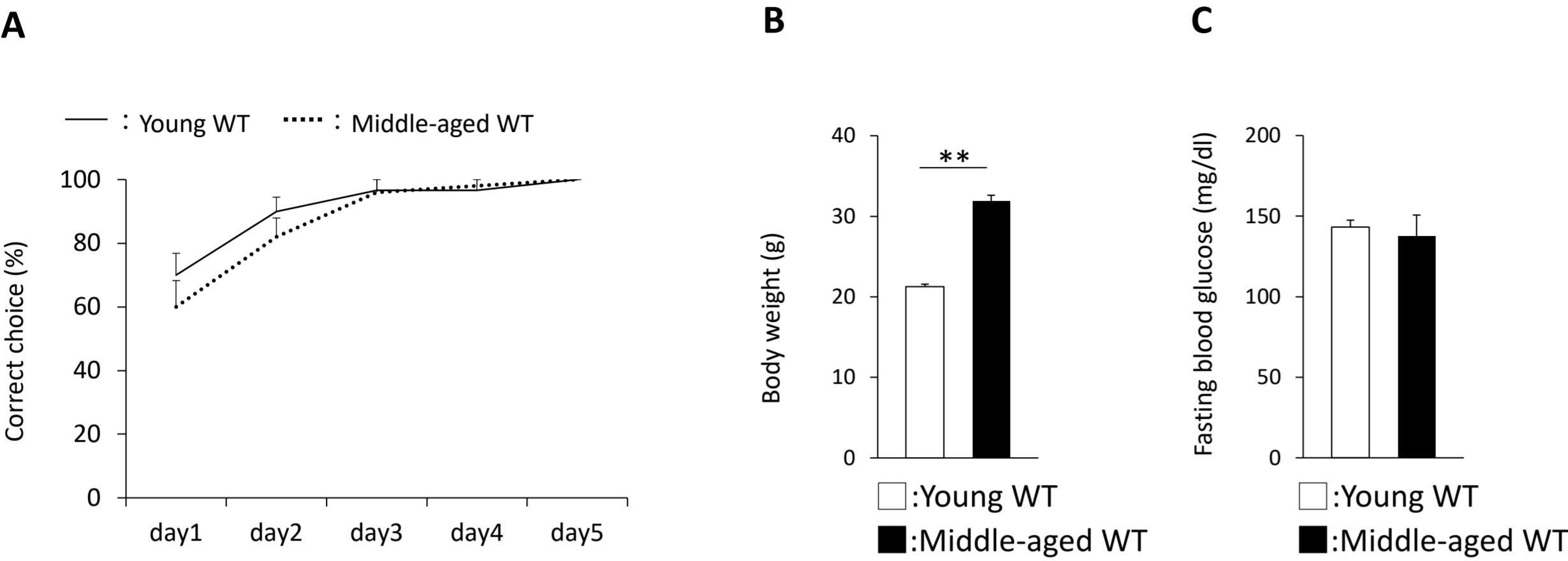

Supplement: Supplementary file 1 — Fig. S1. Middle‐aged WT mice exhibit normal cognitive performance similar to young WT mice regardless of age. [file FEB4-8-1104-s001.pdf]

Supplemental Figure S2.

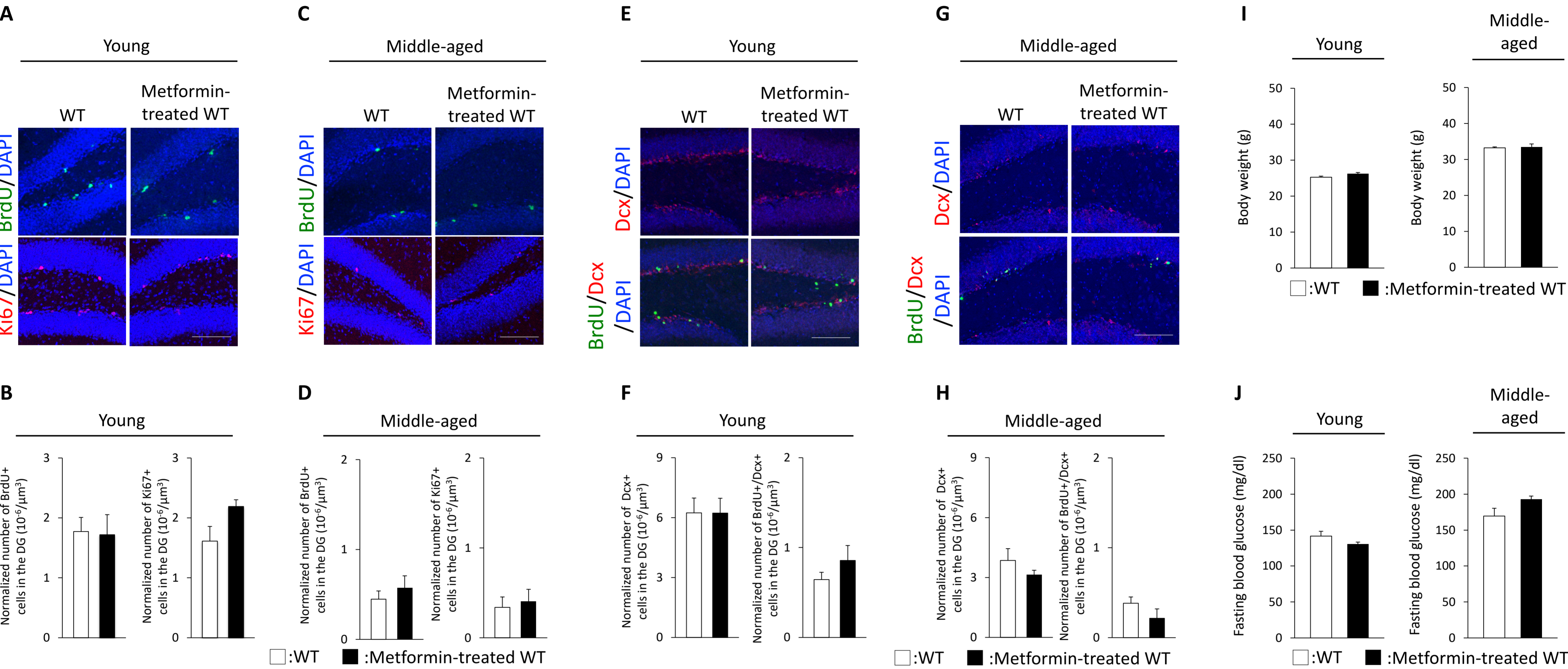

Supplement: Supplementary file 2 — Fig. S2. Chronic metformin treatment has no effect on hippocampal neurogenesis in young and middle‐aged WT mice. [file FEB4-8-1104-s002.pdf]
